# Innovation Pathways for Age-Friendly Homes in Europe

**DOI:** 10.3390/ijerph18031139

**Published:** 2021-01-28

**Authors:** Frans Sengers, Alexander Peine

**Affiliations:** 1 Athena Institute, Vrije Universiteit Amsterdam, de Boelelaan 1085, 1081 HV Amsterdam, The Netherlands; 2 Copernicus Institute of Sustainable Development, Utrecht University, Princetonlaan 8a, 3584 CB Utrecht, The Netherlands; a.peine@uu.nl

**Keywords:** age-friendly homes, innovation, experiments

## Abstract

A variety of innovative pilot projects are being implemented to improve the life-course resilience of existing and newly built home environments. We refer to these projects as “socio-technical experiments” that embody different kinds of promising futures and provide direction to current developments in the emerging domain of age-friendly homes. To take stock of this diversity within Europe; this paper provides an overview of 53 ongoing socio-technical experiments that are being conducted in the Netherlands, France, Ireland and Poland. We find that, besides the variation between European countries, there is a more important type variation in terms of the character of the experiments themselves and the differences in development direction that they propose. Our findings suggest that most of the innovations tested in these experiments are not primarily material or technical but primarily social or conceptual in character (i.e., new organizational modes or everyday practices that re-arrange social relations or new housing concepts that bridge the divide between ageing in place individually and a nursing home). This variety of innovations tested in the experiments can be categorized into seven distinct innovation pathways: (1) Showcasing Technology, (2) Innovation Ecosystem, (3) Sheltered Elite, (4) Specific Community, (5) Conscious Retrofitting, (6) Home Sharing and (7) Retrovation Challenge.

## 1. Introduction: Experimenting with Age-Friendly Homes

The ever-increasing life expectancy of Europe’s urban population is generally considered to be “a great achievement of modern society” [1], p. 2472, and “the culmination of successful human development” [2], p. 733. But the advent of an ageing society also comes with great challenges for the future. One key challenge is that most present-day home environments are not adapted to permit to older adults to age in place. Large parts of the existing building stock are currently not fit for purpose to enable Europeans to lead healthy, active and meaningful lives at home [3]. At the same time, the home is also a site where digital technologies and other novelties are being introduced at an increasing pace and where a wide gamut of innovations meet the muddled realities of the everyday lives of older adults [4]. Therefore, it is crucial to ensure that our homes are suitable and adaptable to our needs and preferences as we age and that they are conducive to integration of promising innovations. In our view, this should be a key part of the response to the demographics of ageing in European cities.

Whilst most of the articles in this special issue deal with the age-friendly *city*, this contribution provides center stage to the smaller scale of the *home*. The first thing that should be noted is that a home is more than a house, apartment or any other physical shell that harbors a domestic living environment. Philosophers, geographers and architects have a long tradition of engaging with the broad concept of home [5,6,7,8]. The notion of home is open to interpretation, sometimes used in a metaphorical way. It is closely related to concepts such as house and dwelling, but it carries with it a set of social and emotional attachments. Home is a “material and an affective space, shaped by everyday practices, lived experiences, social relations, memories and emotions” [8], p. 506, and it is inextricably linked to ideas about “identity, family, nation, a sense of place, and to a sense of responsibility towards who shares this place” [9] and it is a place that offers “security, familiarity and nurture” [6], p. 164. In practical terms for our analysis of innovative activities in the home, this means two things. First, it allows us to focus on social and emotional aspects of a home. These aspects, along with physical elements, together are essential components of what it means to live independently and maintain a good quality life at home [10]. Second, it allows us to look beyond the physical walls of the apartment or house by also including the immediate indoor and outdoor surroundings. Whilst these are not inside the house, they can be included in the wider notion of home. This allows for a focus on a broader set of innovations, not only physical novelties but also social and conceptual novelties—i.e., innovations that do not feature new technology but that feature new ways of organizing social processes or new conceptual housing categories that fill the void between conventional single household apartments and traditional retirement/nursing homes.

Our main concern is thus with the home as a site of *innovation*. Various kinds of organizationally and geographically distinct spaces can be seen as important sites for the concentration and development of particular innovations. These innovative spaces have led to the emergence of new infrastructures, products, activities, services, and industries as well as new sets of user patterns and identities. These spaces include cities, ports, factories, hospitals, offices, but also households or homes. From our perspective as Science and Technology Studies scholars interested in innovation, the home is best described as an “innovation junction”. An innovation junction can be defined as “a space in which different sets of heterogeneous technologies are mobilized in support of social and economic activities and in which, as a result of their co-location, interactions and exchanges among these technologies occur” [11], p. 51. In this context “heterogeneous technologies” refers to a broader conceptualization of technology beyond technical elements and material artefacts; instead, it points to the entire network of technical and social relations [12]. These innovations include not only material novelties (technological innovations) but also novelties in stakeholder roles and social relations (social innovations) and conceptually new ways of doing and organizing (conceptual innovations). An important element of an innovation junction is the co-location of innovation, which encourages certain groups of stakeholders to develop mechanisms and arrangements to coordinate the interaction of these innovations, sometimes through the development of so-called mediating technologies that facilitate and stimulate the interaction between various artefacts and innovative practices. For our purposes, we want to stress that many different kinds of stakeholders are introducing innovations into the homes of older adults. The interactions between the innovations and the coordination attempts between diverse stakeholders are crucial to understand how the material composition, social organization and associated identities of what is considered an “age-friendly home”, are taking shape.

This paper starts from the observation that an increasing number of innovative designs and visionary ideas to improve the life course resilience of existing and newly built home environments are currently being tested in concrete initiatives throughout Europe. We take these initiatives as our starting point and reveal that they represent something more profound. Our aim is to provide an empirical overview and synthesis of these initiatives. As Science and Technology Studies scholars interested in innovation, we have distinct take on these matters, and we hope that the perspective sketched out in this paper provides a meaningful contribution to this special issue. We begin by introducing some key concepts (Section 2). We conceptualize these newly emerging age-friendly homes initiatives as “socio-technical experiments”, and we argue that these experiments are a promising starting point that can lead to wider outcomes in achieving a transition towards age-friendly homes. Next, we present the methodological approach taken to identify and analyze a large set of these experiments (Section 3). We then present the main empirical findings with regard to experimenting for age-friendly housing in different European countries as well as a synthesis (Section 4). A general overview is provided and the specific situation of experimental activity on the ground in four European countries (Poland, France, Ireland and the Netherlands) is sketched out. We also identify a number of general patterns that emerge when we consider this comprehensive set of experiments. Finally, in an effort to synthesize, we articulate a set of “innovation pathways” in order to come to terms with possible futures or potential ways forward. Finally, a reflection is provided on the collection of experiment and the identified innovation pathways, followed by a discussion on the wider support and upscaling dynamics of experiments (Section 5). The paper ends with a brief conclusion (Section 6).

## 2. Theory: Socio-Technical Experiments and Innovation Pathways

We believe that the notion of a “socio-technical experiment” is a promising concept for discussing the relevance of the recent groundswell of age-friendly housing pilot projects. This concept did not originate from the fields of ageing studies or gerontology but from the field of Sustainability Transitions [13]. The Sustainability Transitions literature argues that contemporary environmental problems, such as climate change, present formidable societal challenges. Addressing these problems requires deep structural changes in the systems that provide transport, energy, healthcare, housing and other societal functions [14]. These are called “socio-technical systems” because they entail an array of social and technological elements, including technological artefacts, infrastructures, policies, markets, consumer practices, cultural meanings and scientific knowledge [15]. Changing the structure of an incumbent socio-technical system (called a “socio-technical regime”) requires reconfiguration of the alignment between these elements, and this process starts by experimenting with alternative configurations in a conducive environment that affords a degree of protection from mainstream market selection (called a “socio-technical niche”) [16].

It is important to note that a socio-technical experiment is not the same as a scientific experiment carried out by psychologists or physicists. The laboratory—either as a distinct physical space or as a more general metaphor—sets scientific experiments apart from socio-technical experiments. Scientific experiments are practices that take place in the confines of a laboratory or an otherwise strictly controlled environment in order to find hard objective truths about human nature or material reality. Socio-technical experimentation, on the other hand, implies a more engaged and social constructivist position: society is itself a laboratory and a variety of real-world actors commit to the messy experimental processes tied up with the introduction of alternative technologies and practices in order to purposively re-shape social and material realities [17,18,19].

Socio-technical experiments are thus seen as early expressions of promising new ideas that harbor the seeds of novelty that can contribute to wider socio-technical systems change. Frontrunners or actors who are considered outsiders for incumbent socio-technical systems are often the initiators of these experiments eventually drawing in a broad coalition of actors—from governments and large companies to civil society and grassroots networks of activists. The goal is not simply to establish experimental initiatives as such but to learn about this form of action in the broad sense and find out if the experimental solutions are desirable and effective [16,20]. As the experiment reshapes its institutional environment, fertile ground is created for further chains of experiments and a constituency is built around the alternative socio-technical configuration to form the basis for a new socio-technical system [21]. Formulated in a different way, a socio-technical experiment gives substance to a distinct vision for the future and acts out this future in a small-scale real-life setting. A pilot project with cutting edge smart camera systems and fall detectors in an existing retirement home represents a different possible future as compared to a future represented by a project that demonstrates the feasibility a new form of intergenerational co-housing and formulates this as a challenge to the legitimacy of a traditional retirement home. Since these two very different socio-technical experiments embody distinct future trajectories, they can be said to represent diverging “innovation pathways” for the development of the age-friendly housing niche.

More analytically precise, a socio-technical experiment has been defined by Sengers et al. (2019) as an “inclusive, challenge-led and practice-based initiative designed to promote system innovation through social learning under conditions of uncertainty and ambiguity” [19], p. 161. Let us pick each of the elements of this definition apart. To qualify, an initiative should be *inclusive* in the sense that it is a collective endeavor carried out by a coalition of diverse actors. The *challenge-led* character implies that the initiative is framed as a response to a societal challenge (in our case often related to the pressing need to update the housing with fitting options for older adults). The initiative should be *practice-based* and thus feature a “hands-on” attempt at innovation applied in a real-world setting instead of a laboratory (though sometimes the distinction is not so clear, as in the multitude of “living laboratories” that are now appearing throughout Europe, such as ENoLL, the European Network of Living Labs). The initiative is not simply innovative but should be seen in the context of wider *system innovation*, thus recognizing the material, institutional and cognitive obduracy of the status quo and geared to change key elements of the current way socio-technical systems are configured (in our case systems of housing, care and ICT and their interaction in shaping age-friendly homes). The idea of an initiative promoting *social learning* refers to the way in which stakeholders learn in practice through observation and imitation (important if the experiment is to be followed up) and that they learn in a broad sense; an experiment should allow stakeholders to learn not only about the performance of the innovation itself but also to learn about the wider societal implications of the innovation [21]. The *conditions of uncertainty and ambiguity* in the definition refer to a final feature of these initiatives, namely, that it is unclear to what extent the initiative will attract a wider following in the future (uncertainty), and it is an open question what the desirable and undesirable long-term effects of the intervention will be (ambiguity).

Other scholars have argued for the importance of additional features to characterize a socio-technical experiment. For instance, Vergragt and Brown (2007) talk about “bounded socio-technical experiments” to stress that these initiatives are “bounded” in space and time—i.e., confined within a certain area or community and with a limited duration [22]. The experiments discussed in this paper are bounded by space (the home in the broad sense and experimental site; often territorial boundaries are clear, but sometimes they are more ambiguous), community (older adults or specific sub-groups—e.g., older adults with dementia—are the target audience) and time (sometimes the time during which a new device is being implemented that turns and an existing home into an experimental homes; sometimes the funding duration of a project stipulates the duration, and sometimes there is no clear end for an experiment).

It is important to note that a socio-technical experiment gives substance to a highly distinct vision for the future. Different experiments can be seen as steppingstones toward different futures, which are supported by different values, technologies and institutional arrangements. In other words, experiments can be seen as integral parts of different (competing or complementary) “innovation pathways”. Innovation pathways are journeys that involve key drivers, decision-making junctures, agents and opportunities which combine to facilitate the eventual mainstreaming of a particular socio-technical configuration [23]. Identifying a large number of prominent experiments and articulating a set of corresponding innovation pathways for age-friendly homes might help researchers and practitioner in the domain of age-friendly homes to see the bigger picture and develop a more comprehensive understanding of the innovative activities that they encounter or contribute to.

## 3. Methodological Approach

Our investigation is based on an explorative analysis of socio-technical experiments throughout Europe. Because we set out to collect data about a large number of experiments (our main unit of analysis) within various countries, our research design can be best described as an embedded multiple-case study [24]. Inspired by qualitative methodological approaches for geographers [25], we employ process theory as an explanatory narrative style [26,27]. The approach used for our investigation can be broken down into three separate steps: (1) Scoping, (2) Inquiry and (3) Synthesis.

Our first step of Scoping started with the creation of a database that provides an overview of relevant socio-technical experiments in Europe in the domain of age-friendly homes. To do so we have collected information about projects that pilot test various kinds of innovations related to age-friendly housing. Because we are dealing with socio-technical experiments, we have included not only initiatives that feature technological innovations but also initiatives that feature other kinds of social and conceptual innovations. The experiments that we considered related to age-friendly homes in different ways and brought together elements from multiple logics, rules and institutions (“socio-technical regimes”) that structure the systems of physical housing, healthcare and ICT solutions. Various sources were used to collect experiments. For a start, existing repositories that feature good practices throughout Europe were consulted. When looking for information about the experiments within these digital repositories, more weblinks could be found to other potentially interesting experiments, which were then further explored online through online desk research. Table 1 provides an overview of the repositories initially consulted.

In addition, we contacted several experts from different European countries with a variety of backgrounds (e.g., university, construction, architecture, policy, civil society) who were in a good position to provide additional suggestions for interesting experiments (e.g., university professors specialized in urban ageing, NGOs promoting age-friendly environments, policy network organizations lobbying for the interests of older adults, etc.) When approaching these experts, a clear idea was formulated in terms of the requirements for an experiment to be added to our database. It was communicated to interviewees that we are looking for pilot projects or novel practices, which feature concrete interventions in a specified living environment/home and embody an approach that is considered promising for the future and to an extent innovative (i.e., the projects should contain an element of novelty in terms of either technological/material novelty, such as the introduction of new building materials or supportive ICT devices, or a conceptual/social novelty, such as new busines model for living together or for integrating care into the home environment).

The second step of Inquiry was geared to investigate the experiments and their context in more detail. This required more in-depth information about the realities on the ground in different European countries. Because Europe is diverse, it would be impossible within the scope of this research to obtain a contextualized overview of experiments in each European country. Therefore, the choice was made not to consider the entirety of Europe but instead to zoom in on the following four countries: The Netherlands, France, Ireland and Poland. These four countries were selected for further inquiry in order to take Europe’s diversity into account as much as possible and as a way to compare the situation in different parts of Europe where traditions in their approach to ageing, family structures, care provision as well as the role of the state vary. More specifically, according to Anttonen and Sipilä (1996) each of these four countries represents a distinct “model of social care provision in case of old age” [37]. The Netherlands can be categorized as a part of a “central European subsidiary model” of social care provision in case of old age. In this model the primary responsibility for the care of older adults lies in principle with the family, but the state also plays an important role as steward [37,38]. France can be categorized in terms of a “southern European care model”, which puts even more emphasis on family care [37]. Ireland has been categorized a more “Anglo-Saxon” oriented model of care, which features a smaller role for the state and puts more of an emphasis on means-tested service entitlements [37]. Poland does not feature in the categories devised by Anttonen and Sipilä, and neither does any other Central European country. Whilst far less research has been conducted on this region, we believe it is important to include a country such as Poland in our investigation because it emerged from behind the iron curtain with a different set of institutions related to ageing, care and housing as compared to countries in western and southern parts of Europe.

In each of these four countries, one-week field trip was organized and several socio-technical experiments per country were selected for further inquiry through purposive sampling. The choice for these particular experiments was made because they represent different directions for the future of age-friendly homes and because they were considered pioneering and relevant by interviewed “overview experts” (experts who were in key positions in these countries to provide an overview of important innovative projects; this group includes university professors specialized in urban ageing, NGOs promoting age-friendly environments, policy network organizations lobbying for the interests of older adults, etc.) “Pilot stakeholders” (stakeholders who instigated or were centrally involved in the implementation of the selected socio-technical experiments; this group includes architects, policymakers, founders of NGOs and older adults themselves who are living the experimental realities created by the socio-technical experiments) were also visited and interviewed during the field trips. The questions in the interviews with the pilot stakeholders related to the set-up of the experiment itself (the origin story, milestones, vision behind it, stakeholders involved, what was learned, links to other experiments), the upscaling potential (the kinds of mechanisms that might enable the wider impact of the experiment) and the context and support structures (supportive regulation and other conducive structural elements in a country or region, relations between regimes of housing, care and ICT). A total of 34 in-depth interviews (lasting between 1 and 2.5 h) were conducted with overview experts and plot stakeholders during the field trips. Additionally, seven guided site tours were conducted, which included a guided walk through a pilot apartment building or housing scheme by one of the stakeholders involved in the experiments there.

The third step of Synthesis consisted of a deliberate reflective analysis on the database and country findings in order to compare countries and experiments and to distil patterns and innovation pathways. Patterns here refer to recurring ideas about certain innovations and widely shared reflections that can be found in the stakeholders’ testimonies, and innovation pathways refer to distinct innovation categories into which most of the experiments in each of the four countries can be said to fit. Each category embodies a different promising direction for future development of age-friendly homes in Europe. It is important to note that it is beyond the scope of this paper to provide an account of how the innovation journeys for each of the individual experiments in the database unfolded. But given our efforts, it is within our scope to reflect upon the kinds of future directions that is being constructed as numerous experiments are being carried out. Therefore, our aim in this final step was to provide a synthesis of directions for future development towards which these innovative activities within experiments seem to point and to categorize the innovation activities into a set of distinct innovation pathways. We should note up front that this will not generate the definitive account of innovation pathways for age-friendly housing but rather a first explorative attempt based on the extensive but incomplete information and based on our personal scholarly interpretation.

## 4. Findings: General Overview, National Comparisons, Recurring Patterns and Innovation Pathways

The initial stocktaking exercise resulted in the creation of a preliminary database that contains a large but non-exhaustive collection of relevant experiments related to age-friendly homes Europe. This database was complemented with additional entries during the interviews conducted in the Netherlands, France, Ireland, Poland as the four countries that we analyzed in more detail. To illustrate this, Table 2 below presents several (2 per country) of the 53 innovative socio-technical experiments with age-friendly homes in the four countries. For a full overview of each of the 53 experiments, see Table A1 in Appendix A. The ‘#’ symbol in front of each of the experiment names in Table 2 and Table A1 provides a shorthand identification number. This # number is used in the findings to quickly refer to a specific experiment (e.g. experiment #1 refers to Knarrenhof (Aahof) experiment).

The findings per country in Table 2 allow us to compare a set of experiments for one of the four countries with the other three countries. Combined with additional data collected within these countries, this allows to say something broader about the experimental profile of each country in comparative perspective.

Compared to the other countries studied, the Netherlands features a high percentage of social housing, and the overall quality of the housing stock is high. Certain building design requirements that were considered innovative in some of the other countries are standard practice. In the Netherlands, there are many highly innovative experiments at an operational stage. Some of these are not only new to the country but new to the world. Consequently, a few of these experiments have been widely publicized in international media (for instance, experiment #2 Hogeweyk dementia village and experiment #4 Humanitas intergenerational project) and others are considered curious and interesting by non-Dutch interviewees (for instance experiment #1 Knarrenhof). Some interviewees (both Dutch and foreign) consider the Netherlands a frontrunner in terms of age-friendly housing innovation.

France also shows a wide array of age-friendly home experiments. The broadness of this pallet of activities is exemplified by radically different focus of the two experiments of 27Delvalle (experiment #21) and Maison Babayagas (experiment #22)—the first one is technologically innovative, medicalized and entrepreneurial and the second one conceptually innovative, intentional and community oriented without a focus on new technology or economic development. Many projects could be found in France in the same vein as the 27Delvalle project, building on the synergy between the development of new technologies and associated promises of economic activity. The idea of fostering technological innovation as a starting point for more age-friendly homes and the build-up of an “innovation eco-system” with tech start-ups, larger companies, medical professionals and governments agencies that want to boost this form of economic activity might be a promising approach when it becomes a shared agenda by a relatively powerful groups of stakeholders. In Southern France, there is a longer history of setting up experimental home-like environment setting for medical professionals to conduct experiments that feature advanced technology. There is also a wider palette of projects and initiatives to build up innovation ecosystems as a way to boost local high-value economic activity.

Ireland offers a particularly interesting support context since age-friendly housing and the wider agenda of moving towards an age-friendly society have clearly gained momentum there. This is evident in the large amount of national and local support programmes in line with WHO guidelines, and the attention for Universal Design criteria in many planned projects. Age-friendly housing has been successfully positioned on the political agenda and innovative experiments have emerged as a consequence of this, but the experiments have also contributed to bringing actors together to articulate and empower this agenda in the first place. One thing that plays in the background is the ongoing housing crisis, which has resulted in ideas about freeing up larger family homes and relocating older adults to smaller suitable apartments that have to be built first. Besides many small-scale projects designed with Universal Design criteria, there are also several very innovative experiments such as experiment #23 the Great Northern Haven (technologically innovative) and experiments #24 and #25 on new ways of facilitating home-sharing (conceptually innovative). Overall, in Ireland there is a wide array of different innovative experiments and a willing coalition of housing and government agencies that offer support.

The interviewees in Poland argued that their country is not a frontrunner in terms of age-friendly housing provision and innovation compared to some other European countries. Many of the innovative activities mentioned by the interviewees were considered new to Poland but not new to the world at large. In many cases the interviewees referred to activities in Germany that inspired them or at the difficulties to convince Polish companies that older adults present a growing and viable market for them. Nevertheless, our investigation indicates that in Poland a range of innovative activities are currently gaining momentum, including new intergenerational housing projects and a general push for more apartments dedicated to seniors. Furthermore, the unique experiment #52 Mimo Wieku demonstrator apartment (a seemingly isolated initiative within the Polish age-friendly housing landscape) might be ranked amongst the best designed, high-quality and technologically advanced apartments that were observed during all fieldwork site visits.

If we look at the findings per country, we find differences in innovative practices and in levels of policy support. But the palette of innovative activities for each of the countries is broad and we cannot clearly distinguish France, Poland, Ireland and the Netherlands in terms of ideological pre-occupations. In other words, we do not find clearly distinguishable national-level projections or “socio-technical imaginaries” [39] that specify the nature of innovation agendas and set each country apart in terms of how older adults are portrayed as subjects and in terms of distinct national-level visions for what the future of age-friendly homes should look like. Looking more closely at the “where” of the entire set of 53 experiments—so, not just at the level of the country but at the location of the experiments within each country—we find a dispersed rather than a clustered pattern. This is important because many studies about the geography of innovation and experimentation point to a particular clustering of innovation sites; often around science parks or vibrant larger cities that are home to the “creative class” [40,41,42]. In contrast, if the locations of our 53 experiments would be pinpointed on map for each of the four countries, then a more dispersed pattern would be observed. So, in identifying additional innovative age-friendly home experiments, we should focus our attention not only on booming cities but also on more peripheral towns and villages.

Now let us put national boundaries and locations of experiments aside and consider the focus and directions of the 53 experiments. If the focus of this collection of experiments is in any way representative for the entire array of promising new directions for the future development of age-friendly homes in Europe, then it would seem that most of the associated innovations are not primarily material or technical, but rather social or conceptual in character. These forms of social and conceptual innovation include new organizational modes or everyday practices that re-arrange social relations or new housing concepts that bridge the divide between ageing in place individually and a nursing home. Compared to some of the highly touted technical experiments with smart devices and automated control systems, many of the more radical conceptual innovations with new modes of living together were only identified at a somewhat later stage of the research. The lack of support by powerful business and government coalitions might be a reason why some these conceptual innovations remain under the radar.

For a deeper analysis of innovation activities in experiments, it is crucial to assess the micro processes—or “innovation journeys”—and broader directions—or “innovation pathways” [43]. It is beyond the scope of this paper to provide an account of how the innovation journeys for each of the experiments in the database unfolded (for such an attempt see [44]). But given our efforts, it is within our scope to reflect upon the kinds of future directions that is being constructed as numerous experiments are being carried out. Our aim therefore is to provide a synthesis of directions for future development towards which these innovative activities within experiments seem to point and to categorize the innovation activities into a set of distinct “innovation pathways”. The following seven overarching innovation pathways can be identified:

(1) *Showcasing Technology:* The database of 53 experiments includes many smart home pilots with a high degree of focus on technology. Most of these projects are tangible and clearly defined around solving a problem (though in some cases technology seems to feature as a solution looking for a problem rather than the other way around). These experiments are part of this cluster that provides center stage to new (digital) technology, and they share the idea that better technology makes a better home. Often, technology is showcased in a demonstration home rather than an actual home with a permanent resident. Elements of housing, care and new consumer devices feature in these experiments. In some of these experiments, the demonstration of smart products is more clearly emphasized (e.g., experiment #52 Mimo Wieku, experiment #14 het Slimste huis), and in others, the care component is more dominant (e.g., experiment #23 The great Northern Haven, experiment #12 Belevingswoning Schoneveld)
(2) *Innovation Ecosystem*: Related to the Showcasing Technology pathway above, some of the demonstration homes are also part of a larger agenda to build a regional innovation eco-system around smart home or eldercare technology as their primary goal. In those projects, the demonstration home is not really a home as such since there is no intention of it becoming permanently inhabited by people who can come to call it their place. Examples of experiments in this category include experiment #21 the 27Delvalle and experiment #10 Zorg Innovatie Huis (this is different from the Showcasing Technology pathway above because those apartments are intended to become permanently inhabited by people who will call it their home). Rather than demonstrating to older adults themselves what a future home environment might look like for them, these home-like environments demonstrate technological prowess to investors, healthcare professionals and (to a lesser extent) informal carers. In this case, a building (which might or might not include a home-like demonstrator environment) assumes the role of physical hub to facilitate cooperation between regional stakeholders (such as technology companies, local start-ups, government agencies) and to generate interest amongst other stakeholders that might become enrolled with the eventual goal of strengthening the competitive economic position of the region based on the idea of older adults as a growing market (i.e., a Silver Economy agenda).
(3) *Sheltered Elite*: Also true to the idea of the above Innovation Ecosystem pathway that older adults constitute a lucrative market is the Sheltered Elite pathway. This includes building luxury, high-end sheltered homes designed for older adults who want (and can afford) to live independently with certain well-organized communal facilities and emergency care. It is important to note that this type of housing is not innovative as such, but the reason it is conceptualized here as an innovation pathway is because some of the projects mentioned by interviewees from Poland and to a lesser extent in the other countries included these kinds of housing options when asked about innovative age-friendly housing experiments (e.g., experiment #49 Osiedle Senioralne, experiment #50 Angel Care). Part of the reason for this might be that Poland has a relatively young population that is ageing at a very rapid rate and that still relatively few see this as a potential growth market that is worthy of investment. Apart from issues of growing inequality and whether this would be a desirable pathway in the first place, there is another reason why Sheltered Elite is of interest from an innovation perspective. Compared to more mainstream housing environments, these elite spaces offer an alternative selection environment—or “protective space” [45]—for the development and testing of certain niche innovations.
(4) *Specific Community*: The Sheltered Elite pathway above caters specifically to relatively wealthy older adults, but there are many other examples of experiments directed at other specific sub-groups. Some of experiments feature so-called “intentional communities”, which are deliberately founded for members who hold a common social, political or religious vision and follow an alternative lifestyle. The most well-known of these are religious communities and eco-villages, but the experiment Maison Babayagas for older women with shared feminist principles would certainly also qualify. Interestingly, the international Foundation for Intentional Community views these kinds of collective homemaking arrangements as “pathways towards a more sustainable and just world” [46], to which we might ad that our focus highlights pathways towards an age-friendly world. Whilst specific communities’ highlights similarities amongst residents and the choice to live in a particular way, other interesting experiments highlight different social groups living together sometimes out of necessity rather than choice. Such projects aim to bridge the divides between these groups and deliberately address certain societal problems. A good example is the array of intergenerational housing experiments (a relatively large category in the database of experiments, and examples include experiment #4 Humanitas Deventer, experiment #53 Stalowa-29, experiment #39 Wólczańska-168 and many others).
(5) *Conscious Retrofitting*: Some of the intergenerational housing experiments mentioned above—as well as a large part of the experiments in the database—are located in older buildings with heritage characteristics (e.g., experiment #53 Stalowa-29 and experiment #39 Wólczańska-168). Especially buildings with monument or heritage characteristics exemplify the retrofitting challenges that are associated with making a building as age-friendly as possible on the one hand and retaining features of the original built environment on the other hand. This trade-off has to be made in a deliberate manner, conscious of which criteria are valued over others (hence Conscious Retrofitting). Considerations have to made about how “deep” the retrofit should be and to what extent features full accessibility or renewable energy generation (e.g., experiment #34 Rochestown House) will be taken into account. Finding creative solutions when confronted with an earlier design, choosing which features to retain and which to change presents a very different challenge than building new homes on a greenfield site.
(6) *Home Sharing*: Another interesting conceptually innovative solution that involves deep retrofitting is “home-sharing”. The idea of an older adult living alone renting out a spare room to a student is not new, but what is new is the way that this can now be organized and facilitated (at least that is how many interviewed stakeholders in Ireland perceived the ideas behind experiment #24 Abhaile Project and associated activities by AVA housing). The idea is that a home is adapted to the future needs of the older homeowner and at the same time create rental capacity within this home, which provides financial benefits and a sense of security and community for homeowner and a way to fund the age-friendly retrofit. This process could be outsourced to an intermediary who coordinates and arranges a builder for the physical retrofit, gains access to funding, and selects potential tenants. It might not be a coincidence that this has received more attention in Ireland because of its severe housing crisis, especially in Dublin. Ideas about “rightsizing” (i.e., downsizing) larger homes now inhabited by a single older adult are a point of focus considered both promising and controversial.
(7) *Retrovation Challenge*: A number of experiments have as their main aim to achieve a paradigm shift by fundamentally challenging incumbent institutions and dominant ways of thinking. Other contributors to this special issue have demonstrated how the “responsible rebels” who initiate such experiments have a difficult time navigating an environment with unfavorable selection pressures [47]. What we would like to add is that some of these institutional challengers argue for innovative alternative models that rehabilitate elements from the past in new way. A few new projects challenge the model of institutionalized care whereby older adults become patients instead of citizens (i.e., they are seen as patients dependent of care rather than individuals with their own values, opinions, needs and wants). In the view of these innovators this represents a loss of control and dignity. There is often an explicit agenda to counter stereotypes about older adults as frail and dependent. Some of these projects feature very innovative ideas about rehabilitating arrangements from the past, for example in terms of livable neighborhood design and good neighborship. This recombination of older ideas in a new form that draw inspiration from an imagined past we could call “retrovation”. Interestingly, many retrovation projects are also challengers and vice versa; therefore, these project types have been classified together in name into this “retrovation challenger” category of very innovative conceptual experiments (e.g., experiment #1 Knarrenhof, experiment #2 Hogeweyk, experiment #7 Oudenhuis).

## 5. Discussion

Since our findings are based on a large collection of socio-technical experiments with age-friendly homes, further reflection is required on what this collection of experiments can be said to represent. First of all, it should be noted that even the very notion of the experiment as our main unit of analysis can be scrutinized and that selecting and delineating experiments is not always straightforward. Sometimes it is unclear how an experiment should be delineated exactly and where its borders should be drawn (e.g., experiment #19 is labeled “Andromède intergenerational district & Modu-Lab” because it is not straightforward what should be considered the innovative focal point of the experiment—should it be the life-stage adaptable houses and appartement that make up the larger Andromède intergenerational district, or should it be the specific collective building called Modu-Lab?) Sometimes it is difficult to tie an experiment to a single location or site (e.g., experiment #25 introduces an innovative home sharing platform nationwide; experiment #15 rolls out “pilots sleep-over care” in 10 locations at once; experiment #1 will be replicated in several other locations). Sometimes there are clear cross-links between experiments, even across national borders (e.g., the creation of experiment #17 Alzheimer Village Landais in France was inspired by experiment #2 Hogeweyk dementia village in the Netherlands and lessons between the stakeholders involved were exchanged). Moreover, not all experiments in our database are created equal. A criterion for an experiment to end up in our collection is that it is considered innovative by key stakeholders in within the context of their respective countries. This means that some of the experiments which were considered to be innovative by experts on the ground in some countries might not be considered innovative in other countries (e.g., some of the social housing schemes that were considered innovative in Poland or Ireland would not be considered innovative in the Netherlands).

Second, the procedure for selecting the experiments for the database also deserves further explanation. Our goal has been to engage with innovative experiments throughout Europe, and to do so, we limited our attention to four countries from different parts of Europe with distinct care and housing traditions. This meant, for instance, that we have not looked into the situation in Nordic countries with potentially highly advanced and innovative experiments. The omission of potentially very interesting projects is an inherent risk of our methodological approach, even within the countries where we conducted fieldwork. After all, the existing databases of innovative good practices (such as those of the WHO and various others) yielded a limited number of results and the interviewed helicopters (people in overview positions) and interviewed frontrunners (people who initiate experiments) will have limited knowledge of what kind the innovative activities are being conducted in their countries. Therefore, we cannot claim that the set of experiments that we distilled from this are in any way exhaustive or even representative of the situation. Similarly, the eight pathways that we distilled—based on our own categorization after carefully considering the results from our desktop research, site visits and interviews—are open to interpretation and not definitive as such. Many of the experiments within the database can be considered part of multiple pathways (for instance, there are several housing experiments in Poland which aim to facilitate inter-generational cohousing solutions for people from various age groups, which points to a Specific Community pathway, within old building with heritage characteristics that force the initiators to develop innovative retrofitting approaches to maximized accessibility, which points to a Conscious Retrofitting pathway). Nevertheless, we do believe that our systematic approach has yielded an extensive and comprehensive overview that will be valued by innovators and policymakers throughout Europe who want to learn about innovative practices and concrete initiatives around age-friendly housing.

A key question that we have not addressed in this paper, which is also relevant for innovators and policymakers, is how these experiments can be “scaled up” and contribute to a larger transition to age-friendly home environments. The notion of “scaling-up” is very popular concept amongst policymakers and practitioners who are propagating the uptake of a particular innovation. However, it is also a term that is very analytically imprecise and open to many different meanings [48,49,50,51,52]. Therefore, it might be better to instead speak about the “embedding” of experiments as a way to come to terms with the impact and their wider outcomes. Peine and Arenthorst [3,4] also use the term embedding to highlight wider outcomes of experimentation with age-friendly housing innovations. For them embedding is about mainstreaming innovations through a process by which the innovation becomes internalized into “dominant cultures, structures and practices of everyday life” [4], p. 1325. In our view, the term embedding also entails a reciprocal process as a journey of accumulating changes in relation to cumulatively more ordered and stable socio-technical configurations which experimental outputs come to have an influence. Our notion of embedding thus characterizes the overall process by which outputs of experiments may come to generate wider influence beyond their initial conception and setting, which can happen through a diverse set of mechanisms [53]. This is not limited to the mechanisms providing economic incentives and fostering the creation market for age-friendly homes (as propagated in the Silver Economy narrative). Instead, other activities are crucial as well, such as lesson drawing and reflexive learning around experiments, building a constituency of stakeholders for deeper long-term support, organizing study tours or developing other modes of knowledge exchange to inspire actors elsewhere to set up a similar experiment. For innovation policy, this means that a broader perspective is needed and that a much wider range of activities need to be considered besides financially supporting marketable technologies. This should include finding meaningful ways to support the embedding of the large number of promising experiments with social and conceptual innovations.

If we consider the socio-technical experiment and its wider outcomes as our entry point for achieving a wider transition toward age-friendly housing, then we should acknowledge the relevance of wider “support structures”. By a support structure we mean a more over-arching organizational element that is geared to support concrete experimental activities on the ground. In the literature on transitions and socio-technical experiments, a distinction is made between a “local niche” as collection of individual experiments and a “global niche” as distinct networks of actors “who have some distance to the project, but are related through providing resources, such as finance, political support, technical specifications, that generate a space in which local actors can work” [54], p. 378. Examples of support structures include government subsidy programs, new intermediary organizations around a particular agenda, newly developed standards to promote practices that are not yet mainstream. Consider for instance the case of Ireland, where many interesting experiments are taking place, for which fertile ground has been created by nation-wide initiatives (e.g., National Positive Ageing Strategy aligned with the WHO Active Ageing policy framework and the intermediary organization around the Age-friendly Ireland Programme [55]), various county level initiatives (e.g., the Cúltaca initiative and Older People’s Councils). Vice versa, the experiments themselves also create fertile ground for new and extended support structures. In Ireland, for instance, the Great Northern Haven experiment was important in bringing various stakeholders together in set up some of the above support structures in the first place. Another interesting example of a different kind of support structure is the European Homes4Life project, which has set out to create a Europe wide certification scheme for innovative age-friendly housing solutions [56]. The idea behind this project is that such a new certificate will stimulate investment and eventually make age-friendliness a mainstream criterion to be considered in housing projects (similar to the green building certificates and energy labels that have become mainstreamed over the last years). The process of developing and testing this certification scheme had other effects as well; for instance, initiators of key experiments learned about other experiments elsewhere, and they reflected and re-evaluated some of the features in their experiments.

## 6. Conclusions

Throughout Europe, a variety of innovative socio-technical experiments are being implemented to improve the life-course resilience of existing and newly built home environments. These experiments reflect the distinct socio-economic context of their locations and, more importantly, they provide a glance into potential future directions for the development of age-friendly homes. This paper provided an overview of 53 ongoing experiments in the domain of age-friendly housing in the Netherlands, Poland, Ireland and France as countries that represent different parts of Europe with distinct approaches to housing and care for older adults. Overall, we find that, besides the variation between these countries, there is a more important type variation in terms of differences in the character of these experiments and the directions proposed by these experiments. Most of the associated innovations tested in age-friendly home experiments are not primarily material or technical but primarily social or conceptual in character (i.e., new organizational or everyday practices that re-arrange social relations or new housing concepts that bridge the divide between ageing in place individually and a nursing home). This variety of innovations tested in the experiments has been categorized into seven distinct innovation pathways (Showcasing Technology, Innovation Ecosystem, Sheltered Elite, Specific Community, Conscious Retrofitting, Home Sharing and Retrovation Challenge). We hope that our overview, interpretation and reflection on innovative practices for age-friendly housing and its future directions are a valuable contribution for scholars and practitioners alike.

## Figures and Tables

**Table 1:** Overview of repositories of good and innovative practices related to age-friendly homes (1 August 2020).

WHO Global Database of Age-friendly Practices	55 practices worldwide in the category housing, many of which in Europe [28]
EIP AHA repository	26 practices when searched for the term “home” [29]
EIP AHA refence sites	74 European regions as reference sites, which is more about regional innovation ecosystems rather than concrete experiments [30]
AFE-Innovnet repository	55 initiatives within EU countries, with 12 focusing on housing [31]
European Covenant on Demographic Change repository	showcases a number of initiatives within EU countries, including on housing [32]
PROGRESSIVE project examples of good practices	4 interesting age-friendly housing/environment initiatives across Europe [33]
Ireland’s Age Friendly Cities and Counties catalogue of age-friendly practices	31 projects and organizations in the domain of “housing” in Ireland, as well as other projects and organizations in the domains of “community support and health services” and “communication and information” [34]
Government of Ireland report on Housing Options for Our Ageing Population	12 comprehensive good practices on housing in Ireland [35]
Aedes-Actiz overview of Dutch smart homes for the future	23 smart homes with a care component within the Netherlands [36]

**Table 2:** Overview of 8 key age-friendly home experiments in the Netherlands, France, Ireland and Poland (see Table A1 in Appendix A for the complete overview of 53 experiments).

	Experiment Name	Location	Description
# 1	Knarrenhof (Aahof)	Zwolle 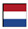	Knarrenhof is an innovative form of housing that actively involves new resident in home making and community support. It is directed at “young older adults” and “old older adults” who want to help each other out and to live independently as long as possible. The attitude and affinity with the neighbors are considered very important, and notions of good neighborship are central. Often those who want to reside here are socially engaged and active (doing voluntary work rather than the stereotype of “bridge clubs and passively sitting at home”) and presented as “social people who can in principle be called upon” by their neighbors. The name “Knarrenhof” consists of two parts. The first part, “knarren” takes it from characters of a popular Dutch TV show from the 1980s (van Kooten en de Bie’s krasse knarren) who are presented as “hardy old geezers” as a way to stress the agency and vitality of older adults (the logo presents an old man showing off his muscles). The second part “hof” can be translated as “courtyard” and refers to the type of picturesque, secluded set of houses facing each other as part urban planning layout conducive for community building. Because this urban form of the courtyard stems from the Middle Ages in Dutch cities and because notions of good neighborship stem from an earlier age, the ideas are presented as “sprung from the past (but) also a project for the future”.
# 2	Hogeweyk	Weesp 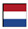	The Hogeweyk a pioneering care facility/community for older adults with dementia. Compared to traditional nursing homes the residents with dementia are more active and live a more “normal” life. Professionally and inhouse trained staff wear regular clothes instead of a uniform and provide the 169 residents the necessary 24-h support in care, living and wellbeing. The “residents, NOT patients” live in one of several housing types that fit their lifestyle (traditional, urban, cosmopolitan and formal—it used to include Indonesian, but this will stop soon because the cohort of older adults from the former colony is getting smaller). The houses of each type are equipped with a shared living room and bedrooms for several (6–7) residents and they are located in a gated neighborhood setting complete with general store, restaurant and theatre (hence the idea of a dementia “village”). The walls are permeable to an extent and people from society outside are encouraged to come in as a way to eventually create a kind of “reverse emancipation” so that society at large becomes more dementia friendly (bringing the outside world in vs bringing the inside world out; social inclusion is a major objective). The underlying vision is to get away from the large-scale medicalized institutionalized model of care home to small-scale normalized social relational model of care.
# 21	27Delvalle	Nice 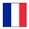	A center on connected health and healthy ageing, which includes a model apartment that is designed as a showcase and a testing platform for technologies supporting independent living and autonomy. The Habitat platform of 27Delvalle is a space dedicated to health and autonomy and facilitates cooperation between a variety of regional stakeholders (Overarching network includes FRANCE SILVER ÉCO, Nice Côte d’Azur Metropolis incubator, CIU Santé, PAILLON2020 and more). It relies in particular on the ecosystem of services dedicated to the loss of autonomy. It prepares the return and promotes the home support of vulnerable people and/or people with disabilities around their personal life project. The “demonstration, simulation and experimentation apartment” is equipped with various digital technologies and innovative devices and is set up to provide advice and solutions to users, their families, caregivers and health professionals. In addition, researchers and industry meet to develop innovative technologies. The objectives are to (1) inform, raise awareness and test; (2) facilitate home return and home support; (3) train medical professionals in new technologies; (4) innovate with research by connecting users, professionals, researchers and industry to be a market access facilitator for businesses.
# 22	La Maison des Babayagas	Paris 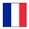	La Maison Des Babayagas is a feminist cohabitation project that started in Montreuil, in the surroundings of Paris in 2013 (Babayagas is a Slavic term for witch). A group of dynamic women have devised a new kind of communal living for older women based on shared values of feminism and activism. La Maison Des Babayagas is a self-managed social housing project composed of 21 apartments for women over 60 and 4 apartments for young adults below 30; the dwellings are still owned by a social housing landlord. Based on four pillars (self-management, solidarity, citizenship and ecology), this “anti-retirement home” aims to facilitate contacts and mutual care between the community members. One of the main motivations for creating the Babayagas house was battling social isolation and many community projects and social activities are organized both by the inhabitants and with the surrounding community, the two rooms on the ground floor of the building being two municipal rooms.
# 23	Great Northern Haven	Dundalk 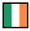	The Great Northern Haven is a new housing project operational for several years now. It features 16 apartments (including one showroom and testing apartment) built to support “life-time adaptability” and Active Assisted Living for older adults. Each apartment is equipped with sensors and interactive technology to support telecare. To an extent, the experiment has been used as a way to convince developers to adopt universal design by making them “walk in the shoes of a frailer older person”. All interviewees in Ireland are familiar with this pioneering high-profile experiment, but according to some of them, the features seem overly hospital-like and heavily reliant on technology. Some of the wiring is now obsolete since smart Wi-Fi solutions were not as prevalent when it was initially designed.
# 24	the Abhaile Project (AVA pilot house)	Dublin 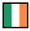	AVA housing offers a solution in the domain of “home sharing”, which offers an alternative to older homeowners whereby their homes are adapted to their future needs whist also creating a rental capacity within their home. This provides financial benefits and a sense of security and community for homeowners. The innovative part is the total package of guidance, support and expertise to the homeowner through the process of retrofitting and sharing arrangements. This particular pilot project put these home sharing into practice in a three-bedroom semi-detached house. The severe housing crisis in Ireland is part of the reason why these kinds of home sharing innovations are gaining momentum.
# 52	Mimo Wieku apartment	Warsaw 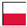	The U Siebie Mimo Wieku (“at home despite the age”) showroom apartment presents a comprehensive set of solutions how to enable older adults to have an active and independent life in their own home. It is the first apartment of this kind in Poland and designed according to best practices regarding accessibility, health, wellbeing and equipped with modern devices to assist older adults and person with disabilities. On about 50 square meters, a well-designed space has been created for a single person or a couple. It is free of physical barriers and ready for upgrades with regard to equipment and amenities. The well thought spatial and physical arrangement, designed by specialized architects, includes solutions with regard to the main aspects of comfort, ergonomics, daylight, illumination and views, indoor air quality, temperature, humidity and air movement and acoustics. The entire array of specially designed building features and smart products make this “larger than the sum of its parts”. Another goal aimed at is to get companies in Poland to see older adults as a relevant market for products and services.
# 53	Stalowa-29	Warsaw 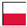	The Stalowa-29 intergenerational apartment building is one of the first cohousing solutions in Poland to be inhabited by people from various age groups. It is a retrofit of an older building (renovation is ongoing at this time) and the idea is that it serves as a model for modern, sustainable and well-designed housing modernization under the Integrated Revitalization Program in the Praga district (it is mostly paid for by city of Warsaw). It is also geared to counter negative effects of gentrification and to encourage residents to help one another. On the last two floors, there will be a care and educational institution for youths. On each of the other floors, 4 apartments are planned (12 in total). On the ground floor a space for the local community will be created in the form of a café or other meeting place (how exactly is yet to be determined). Intergenerational design, countering negative effects of gentrification and encouraging residents to help one another are key elements. According to some interviewees, these kinds of projects are difficult to implement in practice because they feature social housing, and legal requirements stipulate that the next person on the waiting list would qualify for the apartment. Selecting people deliberately based on age and skipping others in line might be hard to justify.

## Data Availability

The first part of the data supporting reported results can be found on publicly available websites and databases (hyperlinks have been provided in the text). The second part of the data supporting reported results are interviews and field visits (interview transcripts, photographs and field visit notes are not publicly available, but can be can be provided by the corresponding author upon request).

## Appendix A

**Table A1:** Full overview of 53 age-friendly home experiments in the Netherlands, France, Ireland and Poland.

	Experiment Name	Location	Description
# 1	Knarrenhof (Aahof)	Zwolle	Knarrenhof is an innovative form of housing that actively involves new resident in home making and community support. It is directed at “young older adults” and “old older adults” who want to help each other out and to live independently as long as possible. The attitude and affinity with the neighbors are considered very important and notions of good neighborship are central. Often, those who want to reside here are socially engaged and active (doing voluntary work rather than the stereotype of “bridge clubs and passively sitting at home”) and presented as “social people who can in principle be called upon” by their neighbors. The name “Knarrenhof” consists of two parts. The first part, “knarren” takes it from characters of a popular Dutch TV show from the 1980’s (van Kooten en de Bie’s krasse knarren) who are presented as “hardy old geezers” as a way to stress the agency and vitality of older adults (the logo presents an old man showing off his muscles). The second part “hof” can be translated as “courtyard” and refers to the type of picturesque secluded set of houses facing each other as part urban planning layout conducive for community building. Because this urban form of the courtyard stems from the Middle Ages in Dutch cities and because notions of good neighborship stem from an earlier age, the ideas are presented as “sprung from the past … (but) also a project for the future”. Aside from the Aahof in Zwolle, there are plans to create more Knarrenhof locations in several other Dutch municipalities.
# 2	Hogeweyk	Weesp	The Hogeweyk a pioneering care facility/community for older adults with dementia. Compared to traditional nursing homes the residents with dementia are more active and live a more “normal” life. Professionally and inhouse trained staff wear regular clothes instead of a uniform and provide the 169 residents the necessary 24-h support in care, living and wellbeing. The “residents, NOT patients” live in one of several housing types that fit their lifestyle (traditional, urban, cosmopolitan and formal—it used to include Indonesian, but this will stop soon because the cohort of older adults from the former colony is getting smaller). The houses of each type are equipped with a shared living room and bedrooms for several (6–7) residents and they are located in a gated neighborhood setting complete with general store, restaurant and theatre (hence the idea of a dementia “village”). The walls are permeable to an extent and people from society outside are encouraged to come in as a way to eventually create a kind of “reverse emancipation” so that society at large becomes more dementia friendly (bringing the outside world in vs bringing the inside world out; social inclusion is a major objective). The underlying vision is to get away from the large-scale medicalized institutionalized model of care home to small-scale normalized social relational model of care.
# 3	Empatisch Wonen	Roermond	59 social housing apartments in a former care home give substance to the vision of “empathic living” (Empathisch Wonen). The main idea behind emphatic living is that a building can be easily transformed to adapt to changing needs of resident groups (i.e., because it is adaptable it “lives with” older adults during their life course, but also for next cohorts of residents such younger people or families). The concept is still relatively open, currently being substantiated. The approach features elements of co-creation and is loosely related to work by visionary Dutch architects from the 1970s. On a secondary level, the emphatic living concept as used in Roermond implies certain features, such as soft walls between one-bedroom apartments (modularity to facilitate future reshuffling of rooms), storage space close to the apartment (for a scoot mobile, but generally useful), a common room (to foster community), broad common hallways (for the scoot mobile and to foster community), lighting solutions for common hallways (daylight to foster experience or solutions with floors that indicate direction) placemaking (has to do with identity and atmosphere and the experience of living), new temperature management, fiber optic internet cabled through a technical room located on the same places on every floor to enable easy access and future smart solutions (to make the home “domotics-ready”), green on balconies (for livability). Aside from Roermond, there are two other locations that will serve as a test site for the Empatisch Wonen concept.
# 4	Humanitas woonstudenten	Deventer	An innovative intergenerational housing arrangement in the Netherlands as an example of how a local long-term older people care practice evolved in response to a national agenda to close down nursing homes in the Netherlands. From 2012 to 2020, there will be 6 students (“woonstudenten”) living amongst the older residents. For a minimum of 30 h a month they are tasked to be a “good neighbor”, for instance by serving bread at the common restaurant.
# 5	Selficient Huis	Utrecht	A self-sufficient modular age-friendly house. Selficient is the name for a housing concept created by a new start-up, and they have a demonstration house in Utrecht, which is portrayed as “the house of the future”. The idea of the Selficient company is to change traditional building practices. Their concept house can be built in a short time through standardized practice. The house is presented as “circular” because sustainable materials are used, can be broken down and rebuilt, and it generates its own energy. Moreover, the house is presented as “modular” because it can be adjusted to “live with you” across the life course. Two specific elements are mentioned as a way to give substance to modularity: future-proof (living as long as possible in the house by adjustments into account in the building process) and life course resilience (house “lives along with you” through the life course since it can be made bigger and smaller with adjustable rooms).
# 6	Woon service zone	Haarlemmer-meer	An innovative home care concept and funding model. A Cristian healthcare foundation and a consultancy and construction management company are developing an affordable “home service zone” (woonservicezone) for various groups in Haarlemmermeer. Such a home service zone is a residential area for 5000 to 20,000 people with many care service facilities and adapted homes. Housing and spatial planning are presented as the “glue” that makes sure that affordable home care is possible. Another interesting associated financial innovation is the idea of an Investment Memorandum as a tool to coordinate investments amongst various kinds of care and construction domain stakeholders involved.
# 7	Ouden Huis	Bodegraven	These 22 apartments provide an alternative for a care home targeted at older adults with or without special care needs. According to the founders “the (traditional) care home strips away your strength and sense of dignity”. the Oudenhuis concept relies on a few key principles related to independence (living on your own and finding company when you want it), aspects of co-living (independent living but aspects of co-living—e.g., shared meals—”so that you know you are not in it alone”), affordability (to include less affluent households), inhouse professional care (through carers in the house, including palliative care), couples stick together (sense of control). Aside from Bodegraven, there are also plans for an Ouden Huis in Woerden and Waddinxveen
# 8	Tuindorp Oost	Utrecht	Since 2016 youth lived alongside older adults in this innovative care home. A stop was introduced which resulted in too many vacant rooms and younger adults looking for housing were allowed to move in. In 2018, it was announced that both younger and older adults had to vacate the place by the end of that year. They were angry about this and did not understand why, especially since this was perceived as a successful experiment. As a result, the younger and older adults banded together to draft a manifesto for better care for older adults. In the manifesto, the younger adults say: “we have a unique perspective on the life of older adults because we have lived amongst them for two years. That is, until [the housing organization] decided to pull us apart too early”. The manifesto is called “give older adults back their voice”.
# 9	De Benring	Voorst	A residential complex with 72 apartments. It was the site of an innovative transformation project which shows how social and technological innovations can be integrated through retrofitting existing real estate for older adults. The built environment is used flexibly, which makes the building “system- and customer preference proof”. De Benring traditional care home was marked for demolition in 2013, but 400 members of the local community spoke out against it and challenged the government. After co-creation workshops they took full responsibility for future functionalities of the building and its prospective future residents. This resulted physically in a refurbished complex (new floor plans and refurbished apartments) with an intergenerational character (10% of the residents being viral youngsters of max 22 years old and 90% older adults of over 55 years old, who learn from each other and help each other out; the youngsters have to take a test to see if they fit and possibly buddy up with an older resident). Various types of home care are also provided, more than possible under normal legal conditions, which effectively safeguards the project against changes in government policy.
# 10	Zorg Innovatie Huis	Baarn	A life course resilient house where older adults, informal carers, healthcare professionals, product and service providers and education stakeholders strive for innovative personalized care. The house provides an inspiring location that collects innovation and new technology for home care and makes it tangible. These smart solutions are tested and learned from in a real world setting with the goal to improve them and to share best practices. The innovations are sub-divided into four groups: (1) physical support, (2) care at a distance, (3) motion, interaction and activation, (4) autonomy and well-being. Examples include smart rollator walkers, smart beds and many robots shaped as small companions or of stuffed animals for cuddling (e.g., Dino Dirk, Maatje, flowerpot Tessa, etc.)
# 11	Het Zorg(T)huis	Winschoten	Project about showcasing technology and giving information; it integrates ideas on smart homes and on care.
# 12	Belevingswoning Schoneveld	Doetinchem	An “experience” apartment showcasing how older adults can live longer at home.
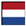
# 13	Huis van Zelfredzaamheid	Enschede	Project about showcasing technology and giving information; it integrates ideas on smart homes and on care.
# 14	Het slimste huis	Alkmaar	Project about showcasing technology and giving information; it integrates ideas on smart homes and on care.
# 15	Pilots logeerzorg	Zeist	The “pilots sleep-over care” are a way to temporarily lift the burden from the shoulder of informal carers. For 10 pilot municipalities the option is given for older adults with high care needs or dementia to temporarily move to a care institution to give some space to informal carers. The programme runs until 2020 and the 10 municipalities are Westland, Capelle a/d IJssel, Dordrecht, Hoeksche Waard, Zeist, Nieuwegein, Helmond, Ede, Heerde and Assen.
# 16	Vivre aux Vignes	Grenoble	A communal living facility that amounts to a novel housing formula with pooled services and care. It is conceptually in between an individual a home and a nursing home and also aimed at older adults with a modest budget.
# 17	Alzheimer Village Landais	Dax	An Alzheimer village in the Southwest of France, inspired by the Dutch project, the Hogeweyk (see above in this table).
# 18	La Note Bleue	Limonest	A residence complex to support ageing in place through adapted housing with 23 units of which 17 are equipped to accommodate people with loss of autonomy.
# 19	Andromede intergenerational district & Modulab	Blagnac	20 houses and 80 apartments are of part of the Andromède intergenerational district, located in Blagnac. The latter is made up of evolving housing that can be adapted to the various stages of life and especially to the loss of autonomy. On this site is also collective building called the Modu-Lab.
# 20	Bailleur social	Lille	Small experiment implementing a modular housing system.
# 21	27Delvalle	Nice	A center on connected health and healthy ageing, which includes a model apartment that is designed as a showcase and a testing platform for technologies supporting independent living and autonomy. The Habitat platform of 27Delvalle is a space dedicated to health and autonomy and facilitates cooperation between a variety of regional stakeholders (Overarching network includes FRANCE SILVER ÉCO, Nice Côte d’Azur Metropolis incubator, CIU Santé, PAILLON2020 and more). It relies in particular on the ecosystem of services dedicated to the loss of autonomy. It prepares the return and promotes the home support of vulnerable people and/or people with disabilities around their personal life project. The “demonstration, simulation and experimentation apartment” is equipped with various digital technologies and innovative devices and is set up to provide advice and solutions to users, their families, caregivers and health professionals. In addition, researchers and industry meet to develop innovative technologies. The objectives are to (1) inform, raise awareness and test; (2) facilitate home return and home support; (3) train medical professionals in new technologies; (4) innovate with research by connecting users, professionals, researchers and industry to be a market access facilitator for businesses.
# 22	La Maison Des Babayagas	Paris	La Maison Des Babayagas is a feminist cohabitation project that started in Montreuil, in the surroundings of Paris in 2013 (Babayagas is a Slavic term for witch). A group of dynamic women have devised a new kind of communal living for older women based on shared values of feminism and activism. La Maison Des Babayagas is a self-managed social housing project composed of 21 apartments for women over 60, and 4 apartments for young adults below 30; the dwellings are still owned by a social housing landlord. Based on four pillars (self-management, solidarity, citizenship and ecology), this “anti-retirement home” aims to facilitate contacts and mutual care between the community members. One of the main motivations for creating the Babayagas house was battling social isolation and many community projects and social activities are organized both by the inhabitants and with the surrounding community, the two rooms on the ground floor of the building being two municipal rooms.
# 23	Great Northern Haven	Dundalk	The Great Northern Haven is a new housing project operational for several years now. It features 16 apartments (including one showroom and testing apartment) built to support “life-time adaptability” and Active Assisted Living for older adults. Each apartment is equipped with sensors and interactive technology to support telecare. To an extent, the experiment has been used as a way to convince developers to adopt universal design by making them “walk in the shoes of a frailer older person”. All interviewees in Ireland are familiar with this pioneering high-profile experiment, but according to some of them, the features seem overly hospital-like and heavily reliant on technology. Some of the wiring is now obsolete since smart Wi-Fi solutions were not as prevalent when it was initially designed.
# 24	the Abhaile Project (AVA pilot house)	Dublin	AVA housing offers a solution in the domain of “home sharing”, which offers an alternative to older homeowners whereby their homes are adapted to their future needs whist also creating a rental capacity within their home. This provides financial benefits and a sense of security and community for homeowners. The innovative part is the total package of guidance, support and expertise to the homeowner through the process of retrofitting and sharing arrangements. This particular pilot project put these home sharing into practice in a three-bedroom semi-detached house. The severe housing crisis in Ireland is part of the reason why these kinds of home sharing innovations are gaining momentum.
# 25	Elder Home Share	nation-wide	New platform that allows older homeowners to continue to live at home with a greater degree of security at night, companionship and help with practical tasks such as light housework and shopping. The other side of the match is a responsible and caring (younger) person who wants an affordable living situation in exchange for sleeping in the house 5 to 6 nights a week and providing 8 h a week companionship and support. The severe housing crisis in Ireland is part of the reason why these kinds of home sharing innovations are gaining momentum.
# 26	Inchicore Housing with Supports	Dublin	Planned housing project with 52 apartments to develop a new model of “housing with supports” for older adults, featuring a physical environment adapted according to universal design principles and appropriate care and supports provided on site, integrated within the local community.
# 27	Broome Lodge	Dublin	43 new apartments built according to Universal Design criteria and rented out social housing by an approved housing body.
# 28	Proudstown	Navan	4 new apartments built on a previously derelict site. Small-scale development that also features renewable energy innovations.
# 29	McAuley Place	Naas	a non-medical, intergenerational and not-for-profit housing association with 53 apartments for social and private housing.
# 30	Colivet Court	Southill	35 apartments designed to be a catalyst in both the social and physical regeneration of the area, generating a sense of pride, empowerment, ownership and mutual respect.
# 31	Leighlinbridge Housing	Leighlinbridge	15 apartments on the grounds of an old presbytery building, providing for security and passive-surveillance and a sense of community.
# 32	Father Lemass Court	Dublin	32 apartments with the goal to create a community through the provision of al central courtyard with an adjoining community room and a communal roof garden, all designed so as to provide passive supervision and social contact.
# 33	SVP Malahide	Dublin	37 apartments devised over wo ranges of housing along opposing sides of the site. This makes the enclosed garden the central focal point, which provides a secure ambience that maximizes passive surveillance and generates an environment of communal engagement.
# 34	Rochestown House	Dublin	34 apartments from the 1970s undergoing a deep energy retrofit to counter fuel poverty and geared to maximize the number of units on this site, which is close to existing services and public transport (this way underused larger council owned houses become available for larger families).
# 35	Glór Na Srútha	Cloncara	12 apartments in a rural village setting and incorporating Age Friendly Design guidelines and universal design principles. Lifetime adaptability, efficiency of technology, and integration with the existing community were all key components (the design responded to site contours and poor ground conditions, and the traditional styles of clustered communities in the locality. The overall design creates a sense of community enclosure through the slow curve of the design whilst retaining its connectivity with adjoining housing scheme through a pedestrian link).
# 36	Cuan an Chláir	Ennis	12 houses and communal facilities. Funding was received from a mix of government funding and other sources based on donations, local fundraising and land allocated by the church.
# 37	Ballygall	Dublin	39 apartments from the 1970s, remodeled, refurbished and energy retrofitted and fully accessible.
# 38	Annamore Court	Dublin	70 newly built social apartments in an existing derelict 1960s social housing scheme with a higher density development (this way underused larger council owned houses become available for larger families). Additional supports and services are provided on site with the aim of supporting independent living in the community for as long as possible. This was the first social housing new-build to benefit from funding provided by the Housing Finance Agency and the European Investment Bank.
# 39	Wólczańska-168	Lodz	This integrational house was partly Inspired by the Warsaw Stalowa-29 exemplar but is now actually at a further stage. This project is about converting an 1883 villa to fit with senior apartments. The project was initiated by seniors from the Forum for the Fatherland Association, who in 2013 submitted their project “Multi-generational House” to the Citizens’ Budget competition where it gained interest among members of the City Council, who in the next year indicated the property at Wólczańska-168 for the Multi-generational House. The funds were secured for a thorough renovation with the adaptation of flats to the needs of the older people, with disabilities (handles in bathrooms, floor showers, no architectural barriers). In 2016, an interdisciplinary team was established within the office, responsible for the development of the Multi-generational House operation program, the work of which in 2018 was supported by the expert team of the Laboratory Foundation for Architecture 60+ as part of a pilot project of revitalization (carried out on behalf of the Ministry of Investment and Development from European funds). The developed model is currently being implemented. Soon the first residents should move in (in accordance with the recommended social mix), an NGO has been running the Neighborhood Club—a place for meetings and integration of future tenants and neighborhood—on the site.
# 40	Inter-generational tenement house Szczecin	Szczecin	A multi-generational house actually in operation. It seems to be a project with senior apartment linked to an orphanage to foster the multi-generation exchange of support (though not much information could be found about this).
# 41	Sheltered housing tenants are waiting for	Ostrów Wielkopolski	14 sheltered apartments were created as part of a larger program in response to the needs of a growing number of seniors and the lack of flats currently dedicated to people 60+ in Ostrów Wielkopolski. Common problems faced by seniors include stairs, no elevator, and a lack of help. This project aims to solve these issues. It is divided into 2 tasks: Sheltered housing Municipal housing Ostrów decided to implement the 1st sheltered flat in order to offer support to seniors who require assistance in everyday functioning but do not have support from their family and do not need service 24/7. It is an alternative to a Social Welfare Home. The Apartments are built in 2 buildings and are adapted to the needs of seniors (lift, wide corridors). They are in the town center, close to a church and a bus stop. The 14 apartments are geared to find out more about the needs of needs older adults (a needs-assessment is conducted).
# 42	Orpea Polska Mieszkania dla seniorów	Wrocław	Housing investment dedicated to older people and independent apartments dedicated to older people with or without assistant needs.
# 43	Orpea stoya rest home	Warsaw	Like the Orpea Polska Mieszkania dla seniorów example above, also a type of alternative nursing home by Orpea.
# 44	Dom dla seniora Szczecin	Szczecin	Dom dla seniora (translated Senior Citizen’s Home) features 15 well-designed apartments (12 one-room units and 3 two-room units). They are located in a building located in the city center of Szczecin, which is equipped with an elevator and designed with older adults in mind, i.e., without architectural barriers.
# 45	Assisted living flats Szczecin	Szczecin	38 assisted living flats for older adults funded local government (though not much information could be found about this).
# 46	Assisted living in Stargard	Stargard Szczeciński	Social housing for older adults with the help of ICT systems and volunteers, the first of this type in Poland. Further search of a WHO database suggests that there are 24 apartments and that this is part of the “house needed” program and the “not alone” program (though not much information could be found about this).
# 47	Mieszkania dla seniorow	Poznan	141 apartments designed exclusively for seniors are located in three buildings. These apartments are intended for older people who have applied for housing in the past but have not received them due to the lack of such a possibility. The apartments became available due to signed collaborations between the city of Poznan and a TBS (a TBS is an institution for a particulate category of semi-social housing).
# 48	Dom Seniora Opole	Opole	102 rental apartments for rent in the TBS Senior system, with 3 buildings of 34 apartments in each. In each building there is a room for shared use by residents (a common room) and facilities for those with mobility impairments.
# 49	Osiedle senioralne	Warsaw	Presented as the First Senior Housing Estate in Poland, which provides an alternative to a nursing home. It is suggested that people feel guilty when they put their parents in a nursing home, but that they should not feel guilty if their parents move to this type of living arrangement. There are 1-bedroom and 2-bedroom apartments available and there is a rehabilitation program to various conditions.
# 50	Angel Care centrum seniora	Wrocław	Angel Care is a nursing home with high-quality nursing support and high-level facilities. This “best nursing home” consist of 48 fully furnished and safe apartments designed for one or two people. In addition. The complex will offer its permanent residents individually designed facilities and tailor-made entertainment and room for their own hobbies. In addition, 24-h nursing, physiotherapy and medical support and specialized beds for medical care are provided. There is a library, common space and workplaces.
# 51	Senioral Apart Hotel Zarabia	Bielsko-Biała	Apartments for older adults on with proximity of mountains and rivers or ski resorts. Their unique selling point is the excellent geographical location.
# 52	Mimo Wieku apartment	Warsaw	The U Siebie Mimo Wieku (“at home despite the age”) showroom apartment presents a comprehensive set of solutions how to enable older adults to have an active and independent life in their own home. It is the first apartment of this kind in Poland and designed according to best practices regarding accessibility, health, wellbeing and equipped with modern devices to assist older adults and person with disabilities. On about 50 square meters a well-designed space has been created for a single person or a couple. It is free of physical barriers and ready for upgrades with regard to equipment and amenities. The well thought spatial and physical arrangement, designed by specialized architects, includes solutions with regard to the main aspects of comfort, ergonomics, daylight, illumination and views, indoor air quality, temperature, humidity and air movement and acoustics. The entire array of specially designed building features and smart products make this “larger than the sum of its parts”. Another goal aimed at is to get companies in Poland to see older adults as a relevant market for products and services.
# 53	Stalowa-29	Warsaw	The Stalowa-29 intergenerational apartment building is one of the first cohousing solutions in Poland to be inhabited by people from various age groups. It is a retrofit of an older building (renovation is ongoing at this time), and the idea is that it serves as a model for a modern, sustainable and well-designed housing modernization under the Integrated Revitalization Program in the Praga district (it is mostly paid for by city of Warsaw). It is also geared to counter negative effects of gentrification and to encourage residents to help one another. On the last two floors, there will be a care and educational institution for youths. On each of the other floors, 4 apartments are planned (12 in total). On the ground floor a space for the local community will be created in the form of a café or other meeting place (how exactly is yet to be determined). Intergenerational design, countering negative effects of gentrification and encouraging residents to help one another are key elements. According to some interviewees, these kinds of projects are difficult to implement in practice because they feature social housing and legal requirements stipulate that the next person on the waiting list would qualify for the apartment. Selecting people deliberately based on age and skipping others in line might be hard to justify.
